# Neutrophil-to-Lymphocyte and Platelet Ratio (N/LP Ratio), a Reliable Criterion for Predicting In-Hospital Mortality in Both Genders Infected With SARS-CoV-2

**DOI:** 10.1155/mi/5720709

**Published:** 2024-12-28

**Authors:** Jafar Mohammadshahi, Hassan Ghobadi, Afshan Shargi, Hossein Moradkhani, Hamed Rezaei, Mahur Kazemy, Mohammad Reza Aslani

**Affiliations:** ^1^Department of Infectious Diseases and Tropical Medicine, School of Medicine, Ardabil University of Medical Sciences, Ardabil, Iran; ^2^Lung Diseases Research Center, Ardabil University of Medical Sciences, Ardabil, Iran; ^3^Department of Community Medicine, School of Medicine, Ardabil University of Medical Sciences, Ardabil, Iran; ^4^School of Medicine, Ardabil University of Medical Sciences, Ardabil, Iran

**Keywords:** coronavirus, COVID-19, sex difference, systemic inflammation indices

## Abstract

**Background:** The neutrophil-to-lymphocyte ratio (NLR), monocyte-to-lymphocyte ratio (MLR), platelet-to-lymphocyte ratio (PLR), derived neutrophil-to-lymphocyte ratio (dNLR), neutrophil-to-lymphocyte and platelet ratio (N/LP ratio), aggregate index of systemic inflammation (AISI), systemic inflammation response index (SIRI), and systemic inflammation index (SII) have emerged as noteworthy determinants in evaluating the severity and mortality prognosis of inflammatory diseases. In order to predict mortality rate, this study aimed to assess the impact of systemic inflammatory markers on both men and women who were admitted to the hospital due to SARS-CoV-2 infection.

**Methods:** The laboratory parameters of the 2007 COVID-19 patients were analyzed in a retrospective study (men = 1145 and women = 862). Receiver operating characteristic (ROC) analysis was used to determine the capability of inflammatory markers to differentiate the severity of COVID-19, while survival probability was determined using Kaplan–Meier curves, with the endpoint being death. To prevent any linear bias, the inflammatory indices were assessed separately using univariate analysis for Charlson comorbidity index (CCI), and adjustments were made for confounding factors if *p* < 0.2.

**Results:** Adjusted-NLR, adjusted-MLR, N/LP ratio, adjusted-dNLR, adjusted-AISI, adjusted-SII, and adjusted-SIRI exhibited remarkably higher values in patients who did not survive as compared to those who did. The multivariate Cox regression models demonstrated significant association between survival and N/LP ratio (HR = 1.564, 95% CI = 1.161 to 2.107, *p* < 0.01) in men and N/LP ratio (HR = 1.745, 95% CI = 1.230 to 2.477, *p* < 0.01) and adjusted-SII (HR = 6.855, 95% CI = 1.454 to 32.321, *p* < 0.05) in women.

**Conclusion:** A reliable predictor in the current study of men and women with COVID-19 was N/LP ratio.

## 1. Background

Although men and women are equally susceptible to COVID-19 infection, men have been reported to have higher incidence and mortality rates than women [1–4]. It is more common for men to encounter the development of infectious diseases, particularly respiratory illnesses and HIV/AIDS [5]. The exact mechanism of sex differences in incidence and mortality rates associated with infectious diseases is unclear. The severity and mortality rates of COVID-19 patients vary due to several key factors, including the following: sex differences in viral entry, sex differences in immune responses, and sex differences in risk factors [6]. In SARS-CoV-2 infection, men have been found to possess higher levels of angiotensin-converting enzyme-2 (ACE2) receptors and transmembrane protease serine 2 (TMPRSS2) compared to women [7–9]. Research involving animals and humans has revealed a direct connection between the expression of ACE2 receptors and TMPRSS2 and the severity of COVID-19 [9, 10]. On the other hand, women had stronger cellular and humoral immune responses than men under SARS-CoV-2 infection, with increased T cell activity [11–13].

Recently, there has been an increasing use of new markers in a variety of inflammatory diseases to estimate severity and mortality rate, which encompass neutrophil-to-lymphocyte ratio (NLR), monocyte-to-lymphocyte ratio (MLR), platelet-to-lymphocyte ratio (PLR), derived neutrophil-to-lymphocyte ratio (dNLR), neutrophil-to-lymphocyte and platelet ratio (N/LP ratio), aggregate index of systemic inflammation (AISI), systemic inflammation response index (SIRI), and systemic inflammation index (SII) [14–19]. Moreover, there is evidence from various studies suggesting the use of systemic inflammation markers to predict mortality rates in COVID-19 patients [20]. Although some studies have reported the efficacy of systemic inflammation markers based on sex in patients with COVID-19, it seems that additional studies are needed. Evaluating the distinctions associated with systemic inflammation markers between men and women infected with SARS-CoV-2 was the objective of this study.

## 2. Method

In a retrospective study, from September to December 2020, patients who tested positive for COVID-19 through a PCR test (Sansure Biotech, China) were recruited for the study carried out at Ardabil Imam Khomeini Hospital in northwestern Iran. The study was conducted after obtaining permission from the Ethics Committee of Ardabil University of Medical Sciences (IR.ARUMS.REC.1400.021).

### 2.1. Data Collection

Comprehensive data were acquired through the electronic medical record system of Imam Khomeini Hospital of Ardabil University of Medical Sciences including parameters such as age, clinical symptoms, medical history, comorbidities, laboratory tests, length of hospitalization, signs, and outcome of the disease (recovery or death). During the initial 24 h in the hospital, diagnostic assessments encompassed a full blood count, assessment of blood clotting, evaluation of kidney and liver function, as well as inflammatory markers such as ferritin, erythrocyte sedimentation rate (ESR), lactate dehydrogenase (LDH), and alkaline phosphatase (ALP).

NLR, PLR, and MLR, as well as dNLR (neutrophils/[white blood cells − neutrophils]), N/LP ratio (neutrophil/[lymphocyte × platelet]), SIRI ([neutrophils × monocytes]/lymphocytes), and SII ([neutrophils × platelets]/lymphocytes), were calculated for all subjects.

The World Health Organization (WHO) guidelines classify the severity of COVID-19 into three levels: moderate for individuals in non-ICU care with severe pneumonia requiring oxygen therapy; severe for ICU patients with mild ARDS; and very severe for those in the ICU with severe ARDS.

### 2.2. Data Analysis

Utilizing SPSS software version 21 and MedCalc version 19.4.1, the data analysis was conducted. For normally distributed variables, mean ± standard deviation (SD) was employed in their presentation, while percentages were used to report categorical variables. Independent group *t*-tests were applied for the purpose of comparing the continuous variables. In order to determine the optimal cutoff values that maximize sensitivity and specificity, receiver operating characteristic (ROC) curve analysis was employed using the Youden index. For survival analysis, time zero was defined as the time of hospital admission. The inflammatory indices obtained from cell counts underwent separate evaluations to avoid any linear bias associated with Charlson comorbidity index (CCI) during the univariate analysis. The CCI is a measure for predicting mortality in patients with a wide range of concurrent diseases (comorbidities), such as heart disease, cerebrovascular disease, chronic pulmonary disease, rheumatologic disease, diabetes, renal disease, malignancy, lymphoma, leukemia, AIDS, peptic ulcer disease, and liver disease, as well as age. A score is assigned based on each of the existing comorbidities, with a score of zero meaning no comorbidity and a higher score meaning a higher probability of mortality in patients. Corrections for confounding factors were also performed if *p*  < 0.2.

The estimation of survival probability was determined for inflammation indices derived from CBC using the means of Kaplan–Meier curves, with the endpoint being death. For both univariate and multivariate analyses, Cox proportional hazards regression was conducted. *p*  < 0.05 was considered significant.

## 3. Results

### 3.1. Demographic Characteristics and Laboratory Parameters

The characteristics and demographics of all patients are presented in Table 1. Out of the 2007 COVID-19 patients included in the study, 1145 (57.1%) were male, and 862 (42.9%) were female. The mean age of female patients (62.78 ± 16.19) was significantly higher than male patients (59.81 ± 17.71, *p*  < 0.001).

Initial investigations of laboratory parameters recorded in patients at the beginning of hospitalization revealed that alanine transaminase (ALT), aspartate transaminase (AST), urea, creatinine (Cr), ALP, blood glucose (BG), ESR, ferritin, and LDH were higher compared to the normal range (Table 1).

Comparing the results of laboratory parameters between genders showed that the levels of Cr (*p*  < 0.001), urea (*p*  < 0.01), ferritin (*p*  < 0.001), AST (*p*  < 0.001), ALT (*p*  < 0.001), international normalized ratio (INR) (*p*  < 0.05), partial thromboplastin time (PTT) (*p*  < 0.01), prothrombin time (PT) (*p*  < 0.05), hemoglobin (Hb) (*p*  < 0.001), and hematocrit (Hct) (*p*  < 0.001) were significantly higher in men than in women. On the other hand, platelet (PLT) (*p*  < 0.001), ESR (*p*  < 0.001), and BG (*p*  < 0.05) levels were significantly higher in women than in men. In addition, women had significantly higher systemic inflammation indices compared to men, such as adjusted-NLR, adjusted-MLR, adjusted-SIRI, adjusted-SII, adjusted-dNLR, and adjusted-AISI (for all *p*  < 0.01) (Table 1).

### 3.2. Clinical Outcomes

The severity of the disease and the mortality rate were not significantly different between men and women. In both genders, based on the outcome of the disease, it was found that the parameters such as age, hospitalization stay, K, ALP, Cr, urea, BG, ferritin, AST, white blood cell (WBC) count, neutrophil count, and monocyte count were significantly higher in the patients who died compared to the subjects who recovered (Table 2). In addition, systemic inflammation indices were significantly higher in both sexes in the deceased compared to the recovered, including adjusted-NLR, adjusted-MLR, adjusted-SIRI, adjusted-SII, adjusted-dNLR, and adjusted-AISI. Interestingly, Cr, ferritin, ALT, INR, PT, Hct, and Hb levels were significantly higher in deceased men than deceased women, while PLT and PLR index were higher in deceased women (Table 2).

### 3.3. ROC

**Figure 1:**
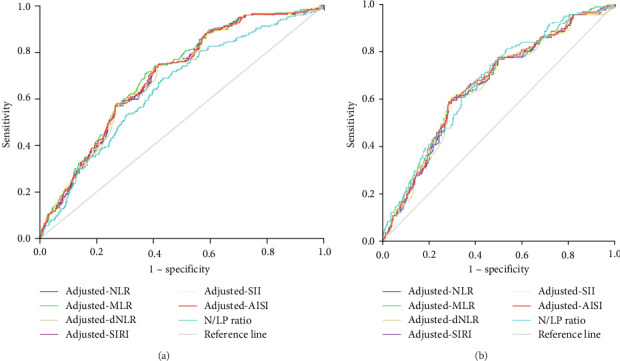
Receiver operating characteristic curve of (A) male patients and (B) female patients for adjusted-NLR, PLR, adjusted-MLR, adjusted-dNLR, N/LP ratio, adjusted-AISI, adjusted-SIRI, and adjusted-SII. AISI, aggregate index of systemic inflammation; dNLR, derived neutrophil-to-lymphocyte ratio; MLR, monocyte-to-lymphocyte ratio; N/LP ratio, neutrophil-to-lymphocyte and platelet ratio; NLR, neutrophil-to-lymphocyte ratio; PLR, platelet-to-lymphocyte ratio; SII, systemic inflammation index; SIRI, systemic inflammation response index. The adjustment is done based on the Charlson comorbidity index.

Based on the ROC curve for survival, optimal cutoff values for systemic inflammatory indices in both sexes were as follows: adjusted-NLR (male = 10.42 and female = 11.21), adjusted-MLR (male = 0.33 and female = 0.33), PLR (male = 255 and female = 247), adjusted-dNLR (male = 4.57 and female = 4.53), N/LP ratio (male = 0.036 and female = 0.032), adjusted-AISI (male = 513,320 and female = 514,124), adjusted-SIRI (male = 2049 and female = 2296), and adjusted-SII (male = 2335 and female = 2339) (Figure 1 and Table 3).

AUC levels in men patients were significant for adjusted-NLR (0.692), adjusted-MLR (0.702), adjusted-dNLR (0.688), N/LP ratio (0.646), adjusted-AISI (0.695), adjusted-SIRI (0.590), and adjusted-SII (0.694) parameters (Figure 1A and Table 3). In distinguishing the dead from the surviving men, it was revealed that the adjusted-MLR had a higher AUC value than the adjusted-NLR (*z* = 2.988, *p*  < 0.01), adjusted-dNLR (*z* = 2.988, *p*  < 0.01), N/LP ratio (*z* = 2.052, *p*  < 0.05), adjusted-AISI (*z* = 2.647,*p*  < 0.01), adjusted-SIRI (*z* = 4.263, *p*  < 0.001), and adjusted-SII (*z* = 2.133, *p*  < 0.05).

AUC levels were significant in women for adjusted-NLR (0.654), adjusted-MLR (0.664), adjusted-dNLR (0.647), N/LP ratio (0.672), adjusted-AISI (0.661), adjusted-SIRI (0.655), and adjusted-SII (0.661) (Figure 1B and Table 3). In distinguishing the dead from the surviving in women, it was identified that the adjusted-MLR had a higher AUC value than the adjusted-NLR (*z* = 2.322, *p*  < 0.05), adjusted-dNLR (*z* = 2.768, *p*  < 0.01), and SIRI (*z* = 3.125, *p*  < 0.01). No significant difference was observed between the two genders in the analysis of AUC levels for systemic inflammation indicators.

**Figure 2:**
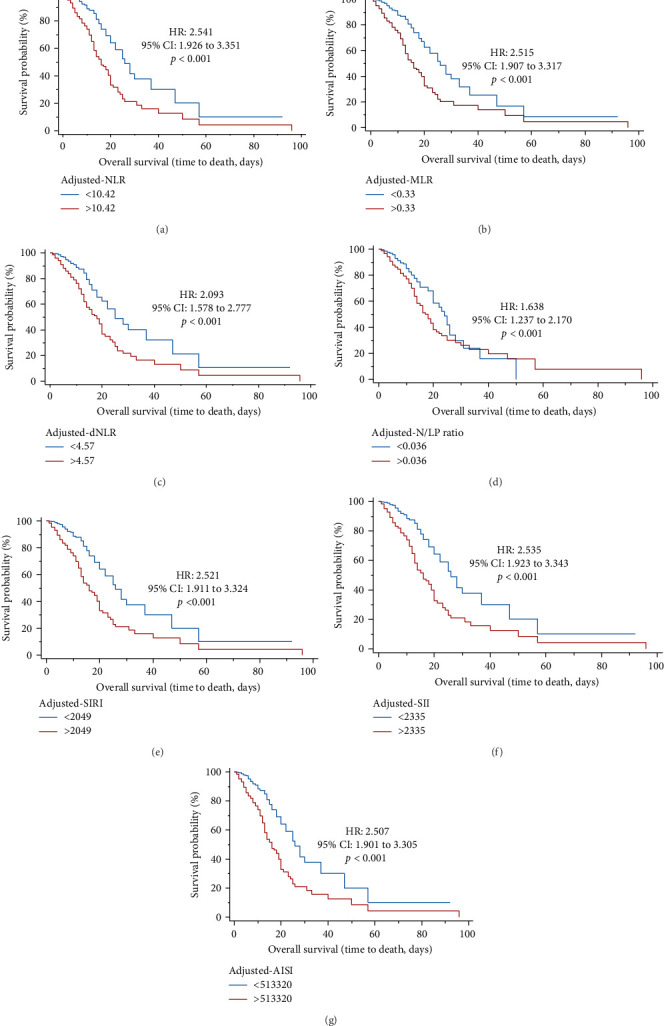
Kaplan–Meier survival curves during hospitalization of male COVID-19 patients with different cutoff values of the systemic inflammation indices investigated. (A) Adjusted-NLR; (B) adjusted-MLR; (C) adjusted-dNLR; (D) N/LP ratio; (E) adjusted-SIRI; (F) adjusted-SII; (G) adjusted-AISI. AISI, aggregate index of systemic inflammation; dNLR, derived neutrophil-to-lymphocyte ratio; MLR, monocyte-to-lymphocyte ratio; N/LP ratio, neutrophil-to-lymphocyte and ⁣^*∗*^ platelet ratio; NLR, neutrophil-to-lymphocyte ratio; PLR, platelet-to-lymphocyte ratio; SII, systemic inflammation index; SIRI, systemic inflammation response index. The adjustment is done based on the Charlson comorbidity index.

According to Kaplan–Meier survival curves, after classifying men patients based on Youden cutoffs obtained with ROC curves, they identified significantly lower survival with higher values of adjusted-NLR (HR = 2.541, 95% CI = 1.926 to 3.351, *p*  < 0.001), adjusted-MLR (HR = 2.515, 95% CI = 1.907 to 3.317, *p*  < 0.001), adjusted-dNLR (HR = 2.093, 95% CI = 1.578 to 2.777, *p*  < 0.001), adjusted-SIRI (HR = 2.521, 95% CI = 1.911 to 3.324, *p*  < 0.001), adjusted-SII (HR = 2.535, 95% CI = 1.923 to 3.343, *p*  < 0.001), adjusted-AISI (HR = 2.507, 95% CI = 1.901 to 3.305, *p*  < 0.001), and N/LP ratio (HR = 1.638, 95% CI = 1.237 to 2.170, *p*  < 0.001) (Figure 2).

**Figure 3:**
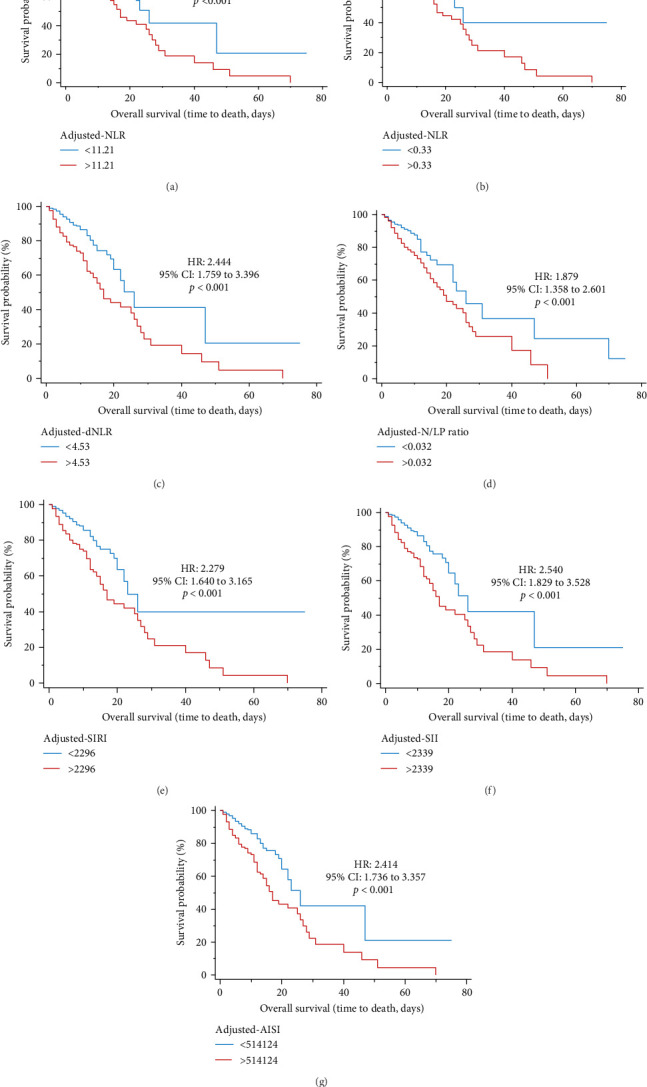
Kaplan–Meier survival curves during hospitalization of female COVID-19 patients with different cutoff values of the systemic inflammation indices investigated. (A) Adjusted-NLR; (B) adjusted-MLR; (C) adjusted-dNLR; (D) N/LP ratio; (E) adjusted-SIRI; (F) adjusted-SII; (G) adjusted-AISI. AISI, aggregate index of systemic inflammation; dNLR, derived neutrophil-to-lymphocyte ratio; MLR, monocyte-to-lymphocyte ratio; N/LP ratio, neutrophil-to-lymphocyte and ⁣^*∗*^ platelet ratio; NLR, neutrophil-to-lymphocyte ratio; PLR, platelet-to-lymphocyte ratio; SII, systemic inflammation index; SIRI, systemic inflammation response index. The adjustment is done based on the Charlson comorbidity index.

On the other hand, in the women, the results revealed that survival was significantly reduced by increasing adjusted-NLR (HR = 2.460, 95% CI = 1.772 to 3.415, *p*  < 0.001), adjusted-MLR (HR = 2.343, 95% CI = 1.689 to 3.252, *p*  < 0.001), adjusted-dNLR (HR = 2.444, 95% CI = 1.759 to 3.396, *p*  < 0.001), N/LP ratio (HR = 1.879, 95% CI = 1.358 to 2.601, *p*  < 0.001), adjusted-AISI (HR = 2.414, 95% CI = 1.736 to 3.357, *p*  < 0.001), adjusted-SIRI (HR = 2.279, 95% CI = 1.640 to 3.165, *p*  < 0.001), and adjusted-SII (HR = 2.540, 95% CI = 1.829 to 3.528, *p*  < 0.001) (Figure 3).

The multivariate Cox regression models in the men showed that only N/LP ratio (HR = 1.564, 95% CI = 1.161 to 2.107, *p*  < 0.01) was significantly associated with survival. On the other hand, in women, it was found that N/LP ratio (HR = 1.745, 95% CI = 1.230 to 2.477, *p*  < 0.01) and adjusted-SII (HR = 6.855, 95% CI = 1.454 to 32.321, *p*  < 0.05) were significantly associated with survival. Interestingly, when both sexes were analyzed in general, it was found that N/LP ratio (HR = 1.482, 95% CI = 1.183 to 1.857, *p*  < 0.001) and adjusted-MLR (HR = 1.340, 95% CI = 1.015 to 1.770, *p*  < 0.05) were significantly associated with survival (Table 4).

## 4. Discussion

The most important findings of the current study were as follows:

1. Although most of the laboratory findings were higher in men than women, the mean age and systemic inflammation indicators were higher in women.
2. Except for PLR, other inflammatory indices were significantly associated with survival in both sexes. In addition, adjusted-MLR had the highest AUC value in both sexes.
3. Based on the results of multivariate Cox regression, it was revealed that only N/LP ratio in men and N/LP ratio and adjusted-SII in women were significantly associated with survival.

Although most studies on COVID-19 patients have reported gender-based differences, some studies have shown no differences [21–24]. Small sample sizes and epidemiological differences have likely influenced these contradictory results [24, 25]. The current study's findings revealed that the percentage of male patients hospitalized was higher than female patients, which was consistent with previous studies [25]. Although the leading cause of sex differences in patients with COVID-19 is not clear, previous studies have also reported a high prevalence of infectious diseases (viral, fungal, bacterial, and parasitic) in men [26]. Other factors involved in sex differences include the role of male sex hormones, predominantly male comorbidities (such as cardiovascular disease and hypertension), age, genetic factors, social factors, obesity, and a weaker immune system in men [1, 27]. In addition, the high distribution of ACE2 receptors in the vital tissues of men compared to women, such as the lungs, heart, and kidneys, may have been another factor in the differences in the COVID-19 incidence [28].

Preliminary evaluations showed that most of the parameters were more severe in men than in women with COVID-19, except for PLT, ESR, and BG. In addition, it was shown in both sexes that most of the parameters (such as leukocyte count, neutrophil count, ferritin, BG, ALP, urea, Cr, and AST) in the deceased were higher than in surviving patients. The results confirmed that the condition was more severe in the dead than in the survivors [6]. Based on these findings, the use of reliable indices in COVID-19 patients can be important in the management and medical care of patients.

Systemic inflammation indices have recently been used to predict mortality in various diseases such as inflammatory diseases and cancer [15, 29]. In COVID-19 patients, several studies have shown their importance in predicting the severity and mortality of patients [15, 20]. In the present study, it was found that women had significantly inflammatory indices such as adjusted-NLR, PLR, adjusted-dNLR, adjusted-AISI, SIRI, and adjusted-SII than men. In addition, in both gender, the systemic inflammation indices (adjusted-NLR, adjusted-MLR, adjusted-dNLR, adjusted-AISI, adjusted-SIRI, and adjusted-SII) were significantly higher in the deceased than in the survivors. In the case of COVID-19, neutrophils and lymphocyte cells appear to play a critical role in host defense against COVID-19, although their role has not been elucidated [30]. Under conditions of COVID-19 infection, the imbalance of T cells occurs, which can lead to a disturbance in the ratio of inflammatory markers such as NLR [15].

AUC and Kaplan-Meier curves were used for the role of systemic inflammation indices in predicting mortality in COVID-19 men and women patients. The results indicated that adjusted-NLR, adjusted-MLR, adjusted-dNLR, N/LP ratio, adjusted-AISI, adjusted-SIRI, and adjusted-SII were in both sexes significantly associated with survival. Interestingly, AUC levels were significantly higher in both sexes about adjusted-MLR. For the first time, based on multivariate Cox regression analysis, the present study results revealed that in women, N/LP ratio and SII remained significant with survival, while in men, only N/LP ratio was significant with survival. Based on systemic inflammation indices at the time of admission, the study results indicate that reliable markers in predicting mortality in both sexes were the N/LP ratio.

Sex differences in the prevalence and mortality of COVID-19 can be explained based on immunological mechanisms, inflammation, and genetic factors. Low mortality rates in women infected with SARS-CoV-2 appear to be due to the effects of estrogen on better regulation of immune responses [31, 32]. Furthermore, T cell production (CD8 T cells) is higher in women infected with SARS-CoV-2 than men [33]. Also, a high concentration of IgG antibodies against SARS-CoV-2 was more evident in women than men [34]. On the other hand, the increase in immunological mediators (IL-16, IL-7, TNFS13B, and CCL23) in men infected with SARS-CoV-2 may have affected the outcome of the disease [35]. Accordingly, biological sex differences may have influenced the pathogenic mechanisms of COVID-19 diseases, such as infection risk, disease severity, and mortality rate.

This study had some strengths and limitations. The strength is the large size. Furthermore, by using multiple hematological indices, this study provided a comprehensive assessment of inflammatory biomarkers. Because this study was only carried out at one institution, it is possible that the results cannot be applied to other healthcare environments. The hematological data were obtained upon admission, which might not accurately reflect how inflammatory markers change over the course of COVID-19 illness. Lastly, the findings may not apply to future patient populations due to the quick advancement of COVID-19 variations.

## 5. Conclusion

The study results demonstrated that gender-based differences in patients infected with SARS-CoV-2 affected systemic inflammation indices in predicting mortality. In men and women, N/LP ratio was a reliable predictor of mortality rate markers, although adjusted-SII in women was also a predictor. Therefore, in COVID-19 patients, as shown by sex-related clinical and laboratory differences, differences in predictors of mortality should also be considered.

## Figures and Tables

**Table 1:** Demographic, hematological, and blood cell count–derived inflammation indices of male and female COVID-19.

Variables	Normal range	COVID-19		*p*-Value
		Male patients (*n* = 1145)	Female patients (*n* = 862)	
Age	—	59.81 ± 17.71	62.78 ± 16.19	<0.001
Hospitalization stay	—	7.34 ± 7.43	7.33 ± 7.11	0.969
WBC (×10^9^/L)	3.5–9.5	7.91 ± 4.56	7.84 ± 4.27	0.758
Neutrophil (×10^9^/L)	1.8–6.3	6.24 ± 3.84	6.02 ± 3.81	0.200
Lymphocyte (×10^9^/L)	1.1–3.2	1.08 ± 1.85	1.14 ± 0.98	0.384
Monocyte (×10^9^/L)	0.2–0.3	0.25 ± 0.19	0.23 ± 0.19	0.184
Hb (mg/mL)	11.5–15	13.81 ± 2.25	12.27 ± 1.96	<0.001
Hct (%)	36–48	40.96 ± 5.98	37.40 ± 5.44	<0.001
PLT (×10^9^/L)	125–350	192.80 ± 87.99	226.14 ± 102.88	<0.001
PT (s)	11–13.5	13.59 ± 3.37	13.23 ± 2.75	<0.05
PTT	30–40	36.20 ± 10.70	34.86 ± 10.38	<0.01
INR	0.8–1.1	1.19 ± 0.62	1.13 ± 0.44	<0.05
ALT (IU/L)	7–40	61.94 ± 138.95	39.26 ± 37.94	<0.001
AST (IU/L)	0–45	78.98 ± 164.69	58.71 ± 47.09	<0.001
LDH (IU/L)	114–240	732.55 ± 357.59	1099.7 ± 1152.4	0.314
Ferritin (µg/L)	11–330	808.17 ± 636.58	449.03 ± 494.27	<0.001
ESR (mm/h)	0–29	40.58 ± 24.01	48.88 ± 23.78	<0.001
BG (mg/mL)	70–100	152.15 ± 87.23	162.93 ± 102.41	<0.05
Urea (mg/mL)	6–24	51.46 ± 41.27	46.41 ± 37.90	<0.01
Cr (mg/mL)	0.5–1.2	1.40 ± 1.23	1.19 ± 0.98	<0.001
ALP (IU/L)	44–147	206.62 ± 144.97	221.46 ± 151.20	<0.05
Na (mEq/L)	135–145	139.48 ± 4.37	139.54 ± 4.42	0.746
K (mEq/L)	3.5–5.3	4.10 ± 0.66	4.14 ± 0.67	0.203
Adjusted-NLR	—	10.73 ± 1.51	10.96 ± 1.43	<0.01
PLR	—	356.11 ± 735.75	414.68 ± 498.56	<0.05
Adjusted-MLR	—	0.32 ± 0.04	0.32 ± 0.03	<0.01
Adjusted-SIRI	—	2147.7 ± 472.22	2217.7 ± 447.52	<0.01
Adjusted-SII	—	2238.7 ± 320.08	2285.7 ± 303.38	<0.01
Adjusted-dNLR	—	4.40 ± 0.42	4.46 ± 0.40	<0.01
N/LP ratio	—	0.13 ± 1.68	0.13 ± 1.28	0.978
Adjusted-AISI	—	483.82 ± 96.04	497.97 ± 90.99	<0.01
Comorbidities, *N* (%)				
Myocardial infarction Heart failure Diabetics Lung disease Cerebral vascular accident Liver disorder Kidney disorder Cancer	—	74 (6.5) 22 (1.9) 278 (24.3) 54 (4.7) 40 (3.5) 15 (1.3) 109 (9.5) 44 (2.8)	54 (6.7) 20 (2.3) 286 (33.2) 47 (5.5) 25 (2.9) 6 (0.7) 63 (7.3) 23 (2.7)	0.440 0.321 <0.001 0.259 0.270 0.131 <0.05 0.168
Charlson comorbidity index	—	2.40 ± 1.90	2.68 ± 1.80	<0.01
Severity				
Moderate, *N* (%) Severe, *N* (%) Very severe, *N* (%)	—	842 (73.6) 113 (9.9) 189 (16.5)	653 (75.8) 61 (7.1) 148 (17.2)	0.087
Outcome				
Survival, *N* (%) Death, *N* (%)	—	934 (81.6) 211 (18.4)	707 (82.0) 155 (18.0)	0.442

*Note:* The adjustment is done based on the Charlson comorbidity index.

Abbreviations: AISI, aggregate index of systemic inflammation; ALP, *alkaline phosphatase*; ALT, alanine transaminase; AST, aspartate transaminase; BG, blood glucose; Cr, creatinine; dNLR, derived neutrophil-to-lymphocyte ratio; ESR, erythrocyte sedimentation rate; Hb, hemoglobin; Hct, hematocrit; INR, international normalized ratio; MLR, monocyte-to-lymphocyte ratio; N/LP ratio, neutrophil-to-lymphocyte and ⁣^*∗*^ platelet ratio; NLR, neutrophil-to-lymphocyte ratio; PLR, platelet-to-lymphocyte ratio; PLR, platelet-to-lymphocyte ratio; PLT, platelet; PT, prothrombin time; PTT, *partial thromboplastin time*; SII, systemic inflammation index; SIRI, systemic inflammation response index; WBC, white blood cell.

**Table 2:** Demographic, hematological, and blood cell count–derived inflammation indices of male and female COVID-19 in survivor and nonsurvivor patients.

Variables	COVID-19			
	Male patients (*n* = 1145)		Female patients (*n* = 862)	
	Survival (*n* = 934)	Death (*n* = 211)	Survival (*n* = 707)	Death (*n* = 155)
Age	57.87 ± 17.72	68.40 ± 14.92^a^	61.16 ± 16.43^b^	70.15 ± 12.30^a^
Hospitalization stay	6.49 ± 5.95	11.09 ± 11.22^a^	6.81 ± 6.00	9.66 ± 10.49^a^
WBC (×10^9^/L)	7.34 ± 3.89	10.40 ± 6.17^a^	7.28 ± 3.68	10.39 ± 5.67^a^
Neutrophil (×10^9^/L)	5.73 ± 3.40	8.50 ± 4.78^a^	5.46 ± 3.20	8.55 ± 5.14^a^
Lymphocyte (×10^9^/L)	1.04 ± 1.23	1.24 ± 3.44	1.14 ± 0.84	1.13 ± 1.46
Monocyte (×10^9^/L)	0.23 ± 0.18	0.28 ± 0.25^a^	0.22 ± 0.02	0.28 ± 0.06^a^
Hb (mg/mL)	13.93 ± 2.16	13.29 ± 2.58^a^	12.31 ± 1.87^b^	12.09 ± 2.31^c^
Hct (%)	41.13 ± 5.66	40.18 ± 7.19	37.41 ± 5.09^b^	37.36 ± 6.82^c^
PLT (×10^9^/L)	193.18 ± 88.39	191.10 ± 86.37	226.22 ± 99.49^b^	225.77 ± 117.45^c^
PT (s)	13.39 ± 3.26	14.38 ± 3.66^a^	13.21 ± 2.88	13.34 ± 2.14^c^
PTT	35.93 ± 10.31	37.27 ± 12.10	34.43 ± 9.20^b^	36.62 ± 14.12^a^
INR	1.16 ± 0.57	1.34 ± 0.77^a^	1.12 ± 0.45	1.17 ± 0.36^c^
ALT (IU/L)	54.53 ± 90.98	91.83 ± 250.83^a^	52.64 ± 34.82^b^	43.36 ± 38.10^c^
AST (IU/L)	66.32 ± 68.52	130.23 ± 339.32^a^	52.64 ± 34.82^b^	84.68 ± 75.59^a^
LDH (IU/L)	675.87 ± 283.89	960.07 ± 514.34^a^	1144.7 ± 1271.7	893.70 ± 444.51
Ferritin (µg/L)	740.77 ± 608.09	1085.43 ± 676.89^a^	370.97 ± 397.20^b^	774.61 ± 690.62^a, c^
ESR (mm/h)	39.21 ± 23.18	47.07 ± 26.94^a^	49.29 ± 23.54^b^	46.86 ± 25.49
BG (mg/mL)	144.44 ± 78.43	186.88 ± 112.94^a^	152.30 ± 87.60	210.66 ± 143.12^a^
Urea (mg/mL)	45.66 ± 34.43	77.24 ± 56.57^a^	41.16 ± 31.02^b^	70.13 ± 53.92^a^
Cr (mg/mL)	1.29 ± 1.06	1.89 ± 1.74^a^	1.11 ± 0.89^b^	1.54 ± 1.26^a (c)^
ALP (IU/L)	199.06 ± 140.74	236.86 ± 156.67^a^	212.70 ± 148.14	259.23 ± 158.87^a^
Na (mEq/L)	139.47 ± 3.91	139.49 ± 6.00	139.73 ± 4.09	138.67 ± 5.64^a^
K (mEq/L)	4.08 ± 0.64	4.19 ± 0.76^a^	4.10 ± 0.61	4.33 ± 0.85^a^
Adjusted-NLR	10.53 ± 1.47	11.64 ± 1.35^a^	10.80 ± 1.41^b^	11.66 ± 1.32^a^
PLR	361.17 ± 800.43	333.75 ± 319.15	399.62 ± 559.63	483.35 ± 748.27^c^
MLR	0.31 ± 0.03	0.34 ± 0.03^a^	0.32 ± 0.04^b^	034 ± 0.03^a^
SIRI	2084.1 ± 45.94	2429.4 ± 42.38^a^	2169.4 ± 440.37^b^	2438.4 ± 413.44^a^
SII	2195.5 ± 31.12	2430.0 ± 28.76^a^	2253.0 ± 298.52^b^	2435.1 ± 280.46^a^
dNLR	4.34 ± 0.41	4.65 ± 0.37^a^	4.42 ± 0.39^b^	4.66 ± 0.37^a^
N/LP ratio	0.13 ± 1.86	0.09 ± 0.13	0.11 ± 1.32	0.19 ± 1.09
AISI	4708.7 ± 934.14	5411.15 ± 862.54^a^	48815 ± 8956^b^	54275 ± 8403^a^

*Note:* Abbreviations are similar to Table 1. The adjustment is done based on the Charlson comorbidity index.

^a^ *p*  < 0.05, in both group survival versus death patients.

^b^ *p*  < 0.05, male survival versus female survival patients.

^c^ *p*  < 0.05, male death versus female death patients.

**Table 3:** Receiver operating characteristic (ROC) curves and prognostic accuracy of blood cell count–derived inflammation indices in male and female COVID-19.

Variables	AUC	95% CI	*p*-Value	Cutoff	Sensitivity	Specificity (%)	*p*-Value (male vs. female)
Adjusted-NLR							
Male Female	0.692 0.654	0.664 to 0.718 0.622 to 0.686	<0.001 <0.001	>10.42 >11.21	74 60	58 70	0.219
Adjusted-MLR							
Male Female	0.702 0.664	0.675 to 0.729 0.631 to 0.696	<0.001 <0.001	>0.33 >0.33	70 60	63 69	0.202
PLR							
Male Female	0.539 0.539	0.510 to 0.568 0.505 to 0.573	0.079 0.151	>255 >247	49 54	63 60	0.996
Adjusted-SIRI							
Male Female	0.692 0.655	0.665 to 0.719 0.623 to 0.687	<0.001 <0.001	>2049 >2296	74 59	58 70	0.221
Adjusted-SII							
Male Female	0.694 0.661	0.667 to 0.721 0.628 to 0.692	<0.001 <0.001	>2335 >2339	73 60	59 70	0.267
Adjusted-dNLR							
Male Female	0.688 0.647	0.660 to 0.714 0.614 to 0.679	<0.001 <0.001	>4.57 >4.53	74 59	58 70	0.189
N/LP ratio							
Male Female	0.646 0.672	0.618 to 0.674 0.639 to 0.703	<0.001 <0.001	>0.036 >0.032	68 65	56 63	0.411
Adjusted-AISI							
Male Female	0.695 0.661	0.667 to 0.721 0.628 to 0.692	<0.001 <0.001	>513320 >514124	73 58	59 71	0.264

Abbreviations: AISI, aggregate index of systemic inflammation; dNLR, derived neutrophil-to-lymphocyte ratio; MLR, monocyte-to-lymphocyte ratio; N/LP ratio, neutrophil-to-lymphocyte and ⁣^*∗*^ platelet ratio; NLR, neutrophil-to-lymphocyte ratio; PLR, platelet-to-lymphocyte ratio; SII, systemic inflammation index; SIRI, systemic inflammation response index; WBC, white blood cell.

**Table 4:** Hazard ratios of the indices under investigation obtained by Cox regression analysis in male and female COVID-19.

Variables	HR	95% CI	*p*-Value
NLR			
Male Female	2.541 2.460	1.926 to 3.351 1.772 to 3.415	<0.001 <0.001
MLR			
Male Female	2.515 2.343	1.907 to 3.317 1.689 to 3.252	<0.001 <0.001
PLR			
Male Female	1.176 1.256	0.891 to 1.552 0.910 to 1.735	0.251 0.164
SIRI			
Male Female	2.521 2.279	1.911 to 3.324 1.640 to 3.165	<0.001 <0.001
SII			
Male Female	2.535 2.540	1.923 to 3.343 1.829 to 3.528	<0.001 <0.001
dNLR			
Male Female	2.093 2.444	1.578 to 2.777 1.759 to 3.396	<0.001 <0.001
N/LP ratio			
Male Female	1.638 1.879	1.237 to 2.170 1.358 to 2.601	<0.001 <0.001
AISI			
Male Female	2.507 2.414	1.901 to 3.305 1.736 to 3.357	<0.001 <0.001

Abbreviations: AISI, aggregate index of systemic inflammation; dNLR, derived neutrophil-to-lymphocyte ratio; MLR, monocyte-to-lymphocyte ratio; N/LP ratio, neutrophil-to-lymphocyte and ⁣^*∗*^ platelet ratio; NLR, neutrophil-to-lymphocyte ratio; PLR, platelet-to-lymphocyte ratio; SII, systemic inflammation index; SIRI, systemic inflammation response index; WBC, white blood cell.

## Data Availability

The datasets used and/or analyzed during the current study are available from the corresponding author on reasonable request.

## Nomenclature

AISI: Aggregate index of systemic inflammation
ALP: Alkaline phosphatase
ALT: Alanine transaminase
AST: Aspartate transaminase
BG: Blood glucose
Cr: Creatinine
dNLR: Derived neutrophil-to-lymphocyte ratio
ESR: Erythrocyte sedimentation rate
Hb: Hemoglobin
Hct: Hematocrit
INR: International normalized ratio
MLR: Monocyte-to-lymphocyte ratio
N/LP ratio: Neutrophil-to-lymphocyte and platelet ratio
NLR: Neutrophil-to-lymphocyte ratio
PLR: Platelet-to-lymphocyte ratio
PLT: Platelet
PT: Prothrombin time
PTT: Partial thromboplastin time
SII: Systemic inflammation index
SIRI: Systemic inflammation response index
WBC: White blood cell.
